# Generic Design of Web-Based Clinical Databases

**DOI:** 10.2196/jmir.5.4.e27

**Published:** 2003-11-04

**Authors:** Jacob Anhøj

**Keywords:** Databases, medical informatics applications, software design, Internet

## Abstract

**Background:**

The complexity and the rapid evolution and expansion of the domain of clinical information make development and maintenance of clinical databases difficult. Whenever new data types are introduced or existing types are modified in a conventional relational database system, the physical design of the database must be changed accordingly. For this reason, it is desirable that a clinical database be flexible and allow for modifications and for addition of new types of data without having to change the physical database schema. The ideal clinical database would therefore implement a highly-detailed logical database schema in a completely-generic physical schema that stores the wide variety of clinical data in a small and constant number of tables.

**Objective:**

The objective was to review the medical literature regarding generic design of clinical databases.

**Methods:**

A search strategy was devised for PubMed and Google to get the best match of peer-reviewed articles and free Web resources on the subject.

**Results:**

Eight peer reviewed articles and a Web tutorial were found. All the resources described the so-called Entity-Attribute-Value (EAV) design as a means of simplifying the physical layout of data tables in a clinical database. In Entity-Attribute-Value design all data can be stored in a single generic table with conceptually 3 columns: 1 for entity (eg, patient identification), 1 for attribute (eg, name), and 1 for value (eg, "Jens Hansen"). To add more descriptive fields to the entity class, all that is necessary is to add attribute values to be stored in the attribute field. The main advantages of the Entity-Attribute-Value design are flexibility and effective entity-centered data retrieval. The main disadvantages are complicated front-end programming needed to display data in a conventional layout that the user understands and less-efficient attribute-centered queries. The Internet offers unique opportunities for database deployment, eliminating problems of user-interface deployment. Furthermore, Web forms may be generated in a completely-generic fashion during run time from metadata describing the semantic structure of clinical information stored in the database.

**Conclusions:**

The Entity-Attribute-Value model is useful for generic design of clinical databases. Depending on the specific requirements of the application, more or less complex metadata models may be applied.

## Introduction

Clinical databases may contain a large variety of data from different domains, eg, patient visits, test results, laboratory reports, diagnoses, therapy, medication, and procedures. Clinical databases may have different purposes, eg, patient management, electronic patient records, clinical research, and quality control. Clinical databases usually have a large number of users with different requirements for views of the database. The administrator does not want to view data per patient, while the nurse must be able to lookup current medication for a specific patient. The researcher may want to do data mining on clinical information for thousands or millions of patients, and the clinician should be able to see his or her ambulatory schedule. Most clinical databases comprise only a part of these functionalities, but these examples illustrate the challenge that designers of clinical databases face. Furthermore, in contrast to schemas from many other domains (eg, finance and public administration) the logical data schemas of clinical data are always incomplete and developing.

In databases, an entity is a single person, place, or thing (eg, patient or diagnostic test) about which data can be stored. In conventional relational database design, each entity is mapped to one or more tables using values of one or more rows to uniquely identify each record. That means that for each entity there exists at least one table. This strategy works well for most databases even if the number of concepts involved in a domain may be high. As long as the domain of interest remains relatively unchanged, the table layout (ie, the physical schema) should work well for many years. The domain of clinical science in particular (and biology in general) is, however, under constant development as new concepts appear and old concepts are modified or deferred.

In a conventional database (that is, in a conventional relational database), new tables must be created to record new concepts. To give users access to the new tables, new forms must be designed and links to these forms must be provided in the user interface. If a table that is already in the database needs to be modified care must be taken not to destroy existing data and not to break any constraints. Accordingly, user-interface forms must be redesigned to reflect changes (eg, fields that have been added or removed) in existing tables.

The complexity and the rapid evolution and expansion of the domain of clinical information thus require a large maintenance overhead if data are laid out using a conventional design. For this reason, it is desirable that a clinical database be flexible and allow for modifications and for addition of new types of data without having to change the physical database schema. The ideal clinical database would therefore implement a highly-detailed logical database schema in a completely-generic physical schema that stores the wide variety of clinical data in a small (and constant) number of tables.

The aim of this project was to provide an overview of techniques and problems in generic design of Web-based clinical databases.

## Methods

Medline was searched through PubMed [1]. Searching was done by trial-and-error using combinations of keywords to get the best match of articles covering the problem. Furthermore a search strategy was devised for Google [2] using a similar trial-and-error strategy.

## Results

The final PubMed search was done on July 11, 2003 using the search term:


(generic database design clinical) OR (entity attribute value).


This term was translated by PubMed into:


(((entity[All Fields] AND attribute[All Fields]) AND value[All Fields]) OR (((generic[All Fields] AND 
("databases"[MeSH Terms] OR database[Text Word])) AND design[All Fields]) AND clinical[All Fields])).


Thirty-three papers were found and 13 were selected based on their title. Of these, 7 were selected based on their abstract and the full-text papers [3- 9] were either downloaded or ordered from the Danish National Library of Science and Medicine.

Google was searched on the same day using the search term:


clinical database generic design.


The search was restricted to the first 30 hits. One additional paper [10] and 1 Web resource [11] of interest were found.

The 9 resources were all from either of 2 research groups: Department of Medical Informatics, Columbia University, New York, NY and Center for Medical Informatics, Yale University, New Haven, Conn. Three production databases were the basis of the 2 group's research: The Clinical Data Repository at Columbia-Presbyterian Medical Center (CPMC), the Adaptable Clinical Trials DataBase (ACT/DB), and SENSELAB.

CPMC [8- 10] is a large clinical repository for millions of patients dating back to the beginning of the nineteen nineties. Several front-end applications offer access to the database giving different views for health care professionals, administrators and researchers.

ACT/DB [3,4,6,7,11] is a clinical-trials database built upon the same design principles as CPMC. Nadkarni et al introduce the term "entity-attribute-value (EAV) design" for generic structuring of data in a relational database [7]. The database is accessible through a generic Web-based interface (WebEAV) [4]. Web forms for displaying and editing data are generated automatically during run time from metadata stored in the database.

SENSELAB [5] is a database for heterogeneous neuronal data. As such it is not a clinical database. However, the SENSELAB architecture uses an object-oriented approach to the EAV model by defining classes and relations (EAV/CR). The EAV/CR architecture is useful for scientific data in general, but it is of special interest for clinical databases.

The principles and design issues involved in these databases are the focus of the remainder of this paper. I will not go into details about the specific implementations of these systems, rather I will present techniques involved in the design of generic database systems. For design details about the 3 database systems the reader is encouraged to consult the references.

### Entity-Attribute-Value Design

In conventional database design, each parameter of interest is represented in a separate column in a table. As new kinds of data need to be managed, the number of columns and/or tables needs to grow.

To add a new attribute for patient description (eg, phone number) to a conventional relational database design (Table 1), another column has to be added to the table.

**Table 1:** Conventional relational database design (example)

PatientID	Name	Date of Birth
1	Jens Hansen	1956-Aug-01
2	Hans Jensen	1974-Sept-04

In EAV design, however, data may be stored in a single table with (conceptually) 3 columns: 1 column for entity identification, 1 for attribute, and 1 for the value of the attribute (Table 2).

**Table 2:** EAV (Entity-Attribute-Value) database design

PatientID	Attribute	Value
1	Name	Jens Hansen
1	DateOfBirth	1956-Aug-01
2	Name	Hans Jensen
2	DateOfBirth	1974-Sept-04

To add a phone number attribute in the EAV table (Table 2), all that is required is to define a new code for phone number to be stored in the attribute column. No change to the table schema is needed. Theoretically, most of the facts that are stored in a database can be stored in a single EAV table.

The EAV design has several advantages:

- **Flexibility:** There are no limits to the number of attributes per entity. The logical database schema can grow without affecting the physical schema.
- **Storage:** In a clinical database thousands of parameters are available while only a few may be recorded for each patient. In a conventional design this may lead to empty (NULL) fields. The EAV design does not need to reserve space for attributes with NULL values.
- **Efficient entity-centered queries:** If, for example, all information for a single patient is needed, it is necessary to query all data tables looking for information about this patient. In a conventional database this may be a time-consuming task that requires looking through hundreds of tables each of which may or may not have information for this patient. As the number of tables and columns grow, the query must be reprogrammed. In an EAV database only 1 table needs to be queried, no joins are necessary, and no change of code is required as the domain evolves. (A join combines data from 2 or more tables based upon a common attribute.)

The EAV design has, however, some drawbacks:

- **Data display:** As discussed later, the user naturally regards data as being organized conventionally in tables and columns regardless of the physical layout of data. Consequently it may be necessary to transform ("pivot") EAV data into a conventional layout when displaying data. This and other tasks that a conventional database would do automatically (eg, referential integrity checking or form-to-subform linkage) require considerable front-end programming in EAV designs. (Referential integrity checking is checking that values in one table that are intended to be used as keys to another table are indeed found in the second table.)
- **Less-efficient attribute-centered queries:** In contrast to entity-centered queries, complex attribute-centered queries, which are based on attribute values, are significantly less efficient and technically more difficult in an EAV database than in a conventional database. The query "show me all patients whose name starts with *J* and whose date of birth is earlier than 1970" is straightforward in a conventional database. To achieve the same result in an EAV database, set operations (for example, INTERSECT) or joins on multiple versions of the EAV table would have to be performed. (INTERSECT is an operation that compares 2 queries to identify records that are found in both.) Set operations and joins are considerably slower than simple select operations. As the number of attributes increase the execution time increases exponentially. Querying EAV data will be discussed in greater detail later.
- **Constraint checking:** In a well-designed conventional database, constraint checking is either unnecessary or trivial. For example, in a conventional table non-null constraints may be placed on columns to prevent incomplete records from being saved. An incomplete record would appear if, for example, the user forgets to fill in a field on a form. In an EAV table a missing attribute-value pair would normally result in a missing record. For example, if no record for one patient's last name is saved in the EAV table this will—from a logical point of view—lead to data that is inconsistent, in the sense that the data for this patient will not be similar to the data for other patients. To prevent this from happening in an EAV database, checking of such constraints should be programmed into the user interface.

### Metadata

EAV design is a way of simplifying the physical schema of the database, making it domain-independent. Regardless of the physical schema, the user naturally perceives the data as conventionally structured in tables and columns. The *logical schema* of the database reflects the user's perception of the data. In an EAV database the logical schema differs greatly from the physical schema. In a conventional database the two are similar. Therefore, an EAV system must have some means of translating the physical schema into a logical schema that reflects the user's understanding of data. This is achieved through metadata (or dictionary) tables whose content defines the semantics of the domain being modeled. An example of a metadata table could be a table listing the attributes available to the data in Table 2. In this example the metadata table would have 2 records, Name and DateOfBirth. If it is necessary to record further information about patients, eg, sex and phone number, that information should simply be added to the metadata table. Thus, in this case, metadata represent what would be the column names of a conventional data table. The metadata model may be enhanced considerably by, eg, adding more descriptive attributes to the metadata table. These attributes may have several purposes—eg, definition of an attribute's data type, constraints, or display layout (text field, select box, etc). These issues will be discussed in greater detail in the next section.

### Evolution of the EAV Model

In the following sections, I give examples of different EAV schemas going from the most-simple, least-flexible to the most-advanced, most-flexible schema. The term "simple" is not to be interpreted as inadequate. The simple solution may be the right solution for a specific task.

The examples reflect the systems described in the literature but are simplified for pedagogical reasons.

#### A Simple EAV Model

**Figure 1:**
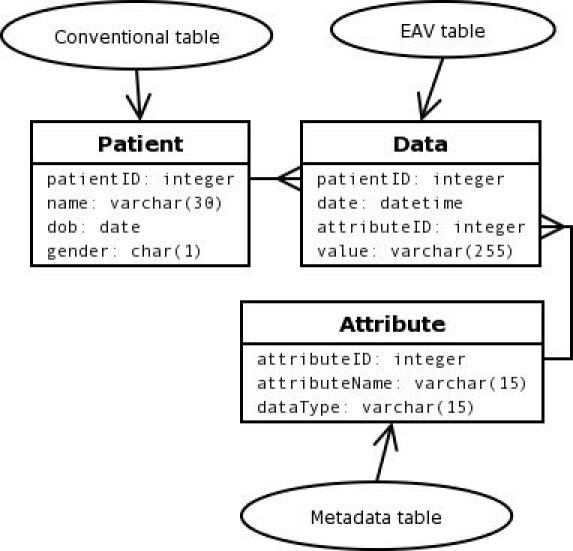
Simple EAV schema for a clinical database. (The crows-foot symbol—3 small lines at the end of a relationship line—illustrates a one-to-many relationship between patient and data, and between attribute and data. Text in each ellipse identifies table type.)

A simple EAV schema for a clinical database is outlined in Figure 1.

Table 3 shows the database tables depicted in the schema of Figure 1. Data have been arranged in a conventional table for patient demographics, an EAV table for clinical events, and a metadata table defining the attributes available to the EAV table. Table 3 represents the patient from Table 1 after a course of influenza that started July 1, 2003 and ended July 11, 2003:

The entity part of the Data table is defined by the combination of patientID and date. The attributeID column holds a reference to the Attribute table, which defines the name and type of available attributes. In a real-world production database there would probably be another table to hold the definition of data types.

Values may of course be of any type, for example, text, number, or Boolean (true/false). In the example in Table 3, the Value field of the Data table is text type. Such a design achieves simplicity by storing all simple types as text values. This approach has, however, some drawbacks. First, not all data types will fit into a text field. Binary objects, eg, x-ray pictures or ECG (electrocardiogram) curves, are too large as are long texts (memo-fields). Second, queries based on values will be less efficient for nontextual values. The text "12" is less than the text "2" even though it is numerically greater, because text is sorted character by character, from left to right.

**Table 3:** Database tables for the simple EAV schema in Figure 1

Patient table*			
patientID	name	Date of Birth	gender
1	Jens Hansen	1956-08-01	Male
			
**Data table**†			
patientID	date	attributeID	Value
1	2003-07-01	1	Influenza
1	2003-07-01	2	2003-07-11
			
**Attribute table**‡			
attributeID	attributeName	dataType	
1	Diagnosis	Text	
2	EndDate	Date	

^*^ Conventional table for patient demographics.

^†^ EAV table for clinical events (data).

^‡^ Metadata table defining attributes available to the EAV table.

Different strategies have been used to store binary data and to increase the efficiency of value-based queries. The simple solution is to ignore the problem and accept that all values be stored as text. This approach may be fully acceptable if it is not necessary to store binary data and if fast value-based queries of large data sets are not required. Another approach is to add a column to the Data table for each data type necessary. For each record, only 1 value-field will be filled in (Table 4).

**Table 4:** Data table with a column for each data type, as a strategy for storing binary objects

patientID	date	attributeID	textValue	numericValue	longValue	dateValue
1	2003-07-01	1	Influenza			
1	2003-07-01	2				2003-07-11

This approach, of course, does not comply with rules for good database design as empty fields are recorded for each record. It may, however, be acceptable in small "quick-and-dirty" applications [12].

The most solid and, from a database designer's perspective, correct solution is to segregate the data table into a number of tables based on the data type of the attribute (Table 5).

**Table 5:** Data table segregated into multiple tables based on the data type of the attribute, as a strategy for storing binary objects

**Data table**		
patientID	date	dataID
1	2003-07-01	1
1	2003-07-01	2
		
**DataDate table**		
**dataID**	**attributeID**	**value**
1	2	2003-07-11
		
**DataText table**		
dataID	attributeID	value
2	1	Influenza

This approach is used in CPMC, ACT/DB, and SENSELAB. For simplicity I chose to show only 1 data table in the illustrations.

The modeling of patient demographic data in a separate conventional table rather than in the EAV table is deliberate (although not necessary). For a schema that is not expected to change often, as is the case with patient demographics, the advantages of an EAV layout do not exceed its disadvantages; and conventional tables and EAV tables can coexist happily together. Furthermore, this design makes it easy to model the one-to-many relation between patient and clinical events. Relations between entities in an EAV table are complicated to model in the simple EAV design. In an electronic patient-record system, for example, it should be possible to record relationships between clinical events (eg, infection leads to a course of penicillin or myocardial infarction leads to death). The enhancement of the EAV design to handle complex relationships between classes will be described later with the EAV/CR schema.

For a simple application intended mainly for data entry, the simple EAV schema may suffice. With the need for a more-advanced user interface for data-display and input purposes, however, some means of grouping attributes becomes necessary. With the simple EAV schema, grouping attributes together on display forms may be done only by entity (patientID and date) or attribute. The application has no way of telling how EAV data records are related and should be displayed together—eg, multiple values from the same blood chemistry panel.

#### Enhancing the EAV Model

**Figure 2:**
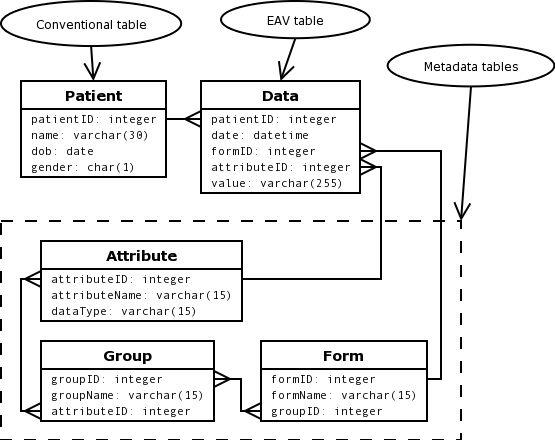
Enhanced EAV schema with grouping of attributes for form display. (Text in each ellipse identifies table type.)

Grouping related attributes for display purposes may be accomplished in several ways. One or more descriptive columns may be added to the "entity part" of the Data table, or the metadata schema may be enhanced. An example of a combination of both methods is shown in Figure 2.

A group table and a form table have been added to the metadata schema. Attributes may now be grouped and attribute groups may be part of forms. To the entity part of the Data table a new field, formID, has been added telling the application to which form a data record belongs. Now any medical event recorded in the Data table belongs to a form and then may be displayed together with all the other attributes on that form. Furthermore, this design facilitates reuse of attribute groups on different forms.

Depending on the domain being modeled and the requirements of the users, other metadata schemas may be suitable.

The simple and the enhanced EAV schemas discussed above are examples of the use of generic EAV tables in clinical database applications. Although to some degree generic, the proposed schemas will need adjustment to the actual domain in question. To achieve total domain-independence more refined models must be created.

#### An Object-oriented Approach to EAV Modeling

The EAV/CR model adds an object-oriented framework to the EAV model by definition of classes and relations. The EAV/CR model was developed for scientific data in general but is useful for clinical data [5].

**Figure 3:**
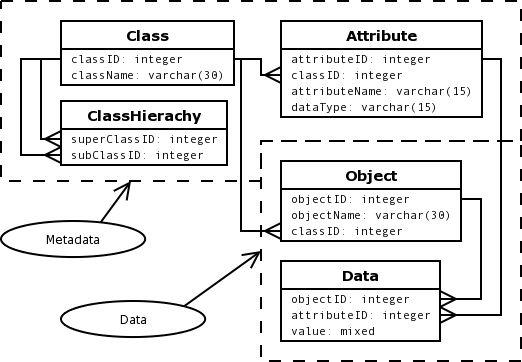
EAV schema with classes and relations (EAV/CR). Simplified from Nadkarni et al [5]. (Text in each ellipse identifies table type.)

Figure 3 shows a simplified example of the EAV/CR table layout used in the SENSELAB database. The class and the attribute tables hold the definitions of classes and their fields. The ClassHierachy table records relations between classes. In this example a subclass can have any number of superclasses, and a superclass can have any number of subclasses. The attribute table records the class to which the attribute belongs and the type of attribute. An attribute can be of any simple type and may even be of class type. Class instances (objects) are recorded in the Object table and instance fields are recorded in the Data table, which is similar to the data table in the simple EAV models.

The example in Table 6 depicts 2 classes, patient and doctor, which are subtypes of a common person class. The patient class has an attribute of object type referring to the patient's responsible doctor. For readability IDs are presented as names instead of numbers.

This example illustrates the use of *inheritance* and *composition* in database design. Inheritance and composition are two important concepts in object-oriented programming. Inheritance can be regarded as an "is-a" relationship between objects—a patient *is a* person, and a doctor *is a* person. Composition is often referred to as a "has-a" relationship—a patient *has a* doctor.

Thus, with this simple layout with (conceptually) just 5 tables, any real-world object can be recorded. Furthermore, objects may be part of other objects; and objects may be related through inheritance. Ad hoc relations between objects (eg, penicillin leads to rash) may be recorded as objects themselves. For this purpose, a class, ObjectRelation, could be defined with 2 attributes, objectID and relatedObjectID. More descriptive attributes may be added to this class if required—eg, causality.

Obviously, considerable up-front programming is required to drive an ergonomic user interface for the EAV/CR model in a real-life production environment. On the other hand, this is a one-time-only job. Another drawback of the EAV/CR design is that the system administrator must have a solid understanding of the object-oriented framework in order to design useful classes. An EAV/CR database is therefore hardly an end-user tool for the average clinician or researcher. As always, flexibility comes with a price.

**Table 6:** Database tables as an example of the EAV schema with classes and relations (EAV/CR) in Figure 3

**Class table**		
className		
Person		
Patient		
Doctor		
**ClassHierachy table**		
superClassID	subClassID	
Person	Patient	
Person	Doctor	
**Attribute table**		
classID	attributeName	dataType
Person	Name	Text
Person	Date-of-birth	Date
Patient	Doctor	Class: Doctor
Patient	Gender	Text
Doctor	Position	Text
**Object table**		
objectName	classID	
Patient01	Patient	
Doctor01	Doctor	
**Data table**		
objectID	attributeID	value
Patient01	Name	Jens Hansen
Patient01	Date-of-birth	1956-08-01
Patient01	Doctor	Doctor01
Patient01	Gender	Male
Doctor01	Name	Doc
Doctor01	Date-of-birth	1960-03-12
Doctor01	Position	Head

#### Querying EAV Data

From a database perspective, querying EAV data is not different from querying conventional data. As mentioned earlier, however, in an EAV database, the physical layout differs greatly from the logical layout, and the user generally wants to see data displayed in a conventional format.

As an example, querying Table 1 for facts about patients whose names start with *Jens* and who were born before 1970 is straightforward:


SELECT * 
FROM table1 
WHERE name LIKE 'Jens%' 
  AND dob < '1970';


To achieve the same result from querying Table 2 requires executing a rather-complex SQL (Structured Query Language) statement:


SELECT d1.patientID AS patientID, 
       d1.value AS name, 
       d2.value AS dob 
FROM table2 AS d1 INNER JOIN table2 AS d2 
  USING (patientID) 
WHERE d1.attribute='name' 
  AND d1.value LIKE 'Jens%' 
  AND d2.attribute = 'dob' 
  AND d2.value < '1970';


The same result may be obtained in several ways, but in any case the query must include set operations (INTERSECT) or as in this example a self join for each attribute. (A self join is a join of a table with itself.) Aside from being complex and out of reach for most end users, these operations are far slower than simple select statements.

I did an experiment using data for one million patients described by 3 attributes: name, date of birth, and gender. These facts were duplicated in a conventional table and in an EAV table in a MySQL database. Three queries were performed on each table with 1, 2, and 3 attributes respectively. Execution time was approximately 2 seconds for the conventional table irrespective of the number of attributes. For the EAV table execution time was 7, 14, and 24 seconds respectively. Thus execution time increases linearly with the number of rows (1 million in the conventional table and 3 million in the EAV table) and—in the EAV table—with the number of joins involved in a query. In the conventional table, however, the number of joins did not affect query time.

Some strategies have been suggested to deal with this problem:

- There may not be a problem. Attribute-centered queries are important for research questions; their performance is not critical for the care of individual patients. If the need for cross-patient data is infrequent the advantages of EAV design probably exceeds the disadvantages.
- Any need for regular cross-patient data access could be met by making backups of the production database and restoring them onto separate hardware. Resource-intensive queries run on the backup data will not affect the production server. Additionally, the EAV data schema could be transformed into numerous conventional tables after backup thus easing query design by end users with modest SQL skills [6].
- If complex, attribute-centered, user-defined, ad hoc queries are important to an application, steps should be taken to facilitate this. First, a user interface, whether graphical or not, should be built to help users retrieve data. The user should be able to freely select any combination of attributes and criteria. The interface should then translate user requests into semantically-valid and syntactically-valid SQL statements; and from the user's point of view, it should not matter whether data are stored in conventional tables or EAV tables. This approach was taken by Nadkarni and Brandt in the development of the ACT/DB Query Kernel [6].
- Optimization of queries may increase the efficiency considerably. Breakdown of complex SQL statements into smaller parts run sequentially may increase query speed. Each part accesses 1 or 2 tables to create a temporary table (or view). These (smaller) temporary tables are then joined [3]. Depending on the ability of the database engine to devise an efficient search strategy, the overall query speed may benefit from creating and joining smaller temporary tables compared to self-joining the full EAV table. An efficient database engine should, however, itself be able to optimize the original query, so that little is gained from this approach. In the MySQL database described above, the creation of a single temporary table took longer (more than 30 seconds) than the execution of the full 3-attribute search (24 seconds).
- Johnson et al [10] suggest an extension to the SQL-query language to facilitate "pivoting" of attribute-centered data into a conventional layout—the Extended Multi-Feature (EMF) SQL. Extended Multi-Feature SQL processing time is linearly proportional to number of attributes.

In summary, querying EAV data is a more complex task than querying data in a conventional layout; and attribute-centered queries are less efficient with EAV data compared to conventional data.

#### Graphical User Interface

The challenge for the user-interface designer of an EAV database is to display data and to let the user manipulate data simulating a conventional layout irrespective of the physical layout—in other words: to bridge the physical and the logical schemas.

The World Wide Web offers an opportunity to simplify database deployment and maintenance. In a typical Web database application, the user's browser requests data from a remote Web server, which sends the request to a database server. After receiving data back from the database server, the Web server formats it into a Web page and sends it to the client browser.

There are several advantages of Web deployment:

- Problems of form deployment are eliminated since all forms reside on the Web server.
- Deployment costs are reduced because Web browsers are available free. Also, hardware costs are reduced since browsers usually have smaller hardware requirements than desktop database-management systems do.
- The form-rendering model of Web pages is simpler and smarter than that of traditional software platforms. Objects on a Web page can be automatically reformatted when the browser win­dow is resized or the user changes the font size. Traditional software developers must put much effort into physical screen size issues. This is not necessary with Web forms.
- Web browsers use clever caching algorithms. That means that when the browser visits a particular page, its contents are cached on the client. On revisit, only components that have changed are downloaded again. This reduces download time and network load.

For these reasons, Web deployment is becoming more and more popular for multi-user applications. However, Web database applications are significantly more complex to develop than traditional database applications for several reasons:

- Web-development tools are less mature than tools for traditional software development; and development of Web database applications still requires much "coding-by-hand." As an example, simple errors such as misspelled variable names, which would be trapped at edit or compile time in a traditional environment, will not be detected until runtime in a Web application.
- Browser-server communication is inherently stateless; when the server has sent a Web page to the client, it "forgets" about the client. Tracking information (eg, user authentication) through several Web pages therefore involves extra programming. To maintain information, the developer must store data either in (hidden) form fields on Web pages or in session variables, which can be accessed as long as the session lasts. Both approaches complicate development and may compromise security because other users (or processes) may gain access to these data intentionally or accidentally.
- Designing Web forms requires much more programming than does designing forms in traditional client-server environments. Web form fields are typeless and input masks for formatting user inputs are not inherent parts of Web forms. (In typeless fields the user may accidentally enter numbers in text-only fields or accidentally enter text in numbers-only fields.) This puts pressure on the programmer to put much effort into both client-side and server-side data validation. In a traditional environment, form fields may be typed; thus, eg, the programmer does not need to worry about users entering letters in number fields or invalid dates in date fields. In a Web form, all validation procedures must be hand coded. Finally, population of select boxes (drop-down menus) and radio buttons (option buttons) with dynamic data is usually much easier in a traditional environment than on a Web form.

Programming Web forms is tedious and error prone, and automation is highly recommended. Nadkarni et al have studied a generic framework for automatic generation of Web forms for display and manipulation of EAV data (WebEAV) [4]. The main objective was to automate the generation of Web forms based on metadata in an EAV database. When details about an event are requested, a form is generated from the metadata of the attributes involved. Each form field has a unique name, which is constructed such that the field name contains its own metadata. When data is sent back to the server, the server creates the correct SQL statements by parsing field names, and data are updated accordingly.

WebEAV makes extensive use of client-side validation of data. Standard validation code in the form of JavaScript is built into the Web page. Validation relies on the use of form field events (eg, OnChange, OnFocus, and OnBlur) and metadata for the attributes in the form (eg, data type, maximum and minimum bounds, and non-null requirements).

## Discussion

Based on searching the literature, it appeared that the Entity-Attribute-Value model is useful for generic design of clinical databases. The most advanced model uses an object-oriented approach and gives tremendous flexibility, allowing the designer to model any type of concept and any relation between concepts in the domain of interest without ever having to worry about changing the table layout or maintaining the user interface. With the ever changing and evolving domain of clinical information, generic design is of special interest for clinical databases, because changes to the logical schema will not affect the physical schema. However, database designers from other areas (eg, biology or literature) may also find the EAV approach useful.

Historically, EAV was introduced into clinical databases in TMR (The Medical Record), built at Duke in the 1970s [13,14]. In addition to the ones mentioned in this paper, production databases using EAV components include TrialDB [14], the HELP system [15], the Cerner and 3M repositories, ClinTrial, and Oracle Clinical.

### Pros and Cons of EAV Design

The advantages of generic design are obvious. The disadvantages, however, may be less obvious and depend on the objectives of the specific application in question.

From a performance point of view, the strength of the EAV design lies in effective entity-centered queries since no joins are necessary to retrieve all facts about entities (eg, patients or medical events) as would be the case in a conventional design with facts spread over hundreds of tables. The drawback lies in inefficient attribute-centered queries, since a (self) join is necessary for each attribute that is requested.

Performance of EAV tables may not be an issue for small databases, but for large clinical repositories with hundreds of concurrent users, query time may be a critical factor. Also, the need for complex attribute-centered data retrieval differs greatly between applications. An electronic patient-record system, for example, is usually aimed at displaying patient-centered (ie, entity-centered) facts, while a research database usually must have some means of aggregating data across a large number of patients. In the latter, however, query efficiency may not be a problem, since data summaries are retrieved only intermittently and may be stored on separate hardware.

These issues warrant careful design of the database schema and cautious decisions about when to use conventional tables in place of generic EAV tables. As a rule of thumb, conventional table design is appropriate for entities whose schemas are not expected to change often (eg, people or institutions).

### Metadata Preserves Information

The simplicity and flexibility of the database schema also increases the complexity of collecting and displaying information from data. The user needs to see and enter related data on the same form. Often single values do not make sense unless coupled with other values. Take as an example a body weight of 176 lb (80 kg). This would be perfectly normal (and desirable for some of us) for an adult male with a height of 5 ft, 10 in (182 cm). For a 10-year-old girl, 176 lb would be highly disturbing. Using a simple EAV data table layout, relations between data are lost unless steps are taken to store these as well. This is the whole idea of metadata—to conserve information about relationships between atomic data values. The metadata schema is the only thing that differs between the different EAV models presented in Figure 1, Figure 2, and Figure 3 and between the actual implementations of the EAV model presented in the articles. The data parts are for practical purposes the same.

It appears that metadata schemas themselves may be more or less generic depending on how closely related they are to the actual domain being modeled. The more specific the metadata schema is, the less flexible it will be. On the other hand, a specific metadata schema will require less programming to drive the user interface than a highly generic one.

To summarize this part, the choice of model depends on the domain and the requirements for flexibility. The object-oriented approach is by far the most flexible solution and in many ways an elegant solution. On the other hand, the complexity introduced by this model may not be justified unless the domain requires the fine-grained control over objects and relations. A simple model may well be the right solution for a simple job.

### Databases and Objects

Much effort has been put into generalizing clinical databases. The most flexible and generic models take an object-oriented approach to data modeling the mapping of objects to tables in a relational database. There is no doubt that object-oriented design is "hot" in the medical area. But porting of object-oriented generic databases from traditional relational databases to produce object-oriented database management systems (OODBMSs) does not seem to be just around the corner. One reason for this may of course be that object-oriented database management systems are still lagging behind relational database management systems with respect to efficiency and availability, although extensive research is going on in this field. Furthermore, object orientation is still a new concept to most clinicians who design databases. But even with modest skills in an object-oriented programming language such as Java, the similarities between object-oriented programming and object-oriented data management seem striking.

Object-oriented databases come in two flavors [16]:

- Systems that provide object-oriented extensions to relational systems by adding composite attributes, class hierarchies, and extensions to a data manipulation language such as SQL. These systems are called *object-relational* systems.
- Systems that extend an existing object-oriented programming language like C++ or Java to deal with databases. Such languages are called *persistent* programming languages. The term "persistent" refers to the fact that the programming language must devise some means of storing objects even when the program is not running. Databases built upon persistent programming languages are called *object-oriented* databases.

The former approach has similarities to the approaches described in this project in that these build upon conventional relational database management systems. The SENSELAB database allows for composition and inheritance, and CPMC has explored the extension of SQL to facilitate attribute-centered querying EAV data.

The latter approach to *generic* database design has to my knowledge not been described in the medical literature. The idea of encapsulating all data and functionality relevant to an object within each object opens up a plethora of possibilities of interest for the developer and manager of clinical information systems:

- The object-oriented paradigm ("everything is an object") is a means of describing real-world concepts, and objects may be easier to understand for a clinician than complex relationship sets in a relational database. One could say that object-oriented design brings together the logical and the physical schema. Even if this may not be completely true, the user should not have to worry about how to design tables for storing of objects. The database will take care of this.
- Object-oriented languages handle complex attributes and inheritance much more elegantly than do even the most cleverly-designed relational database. When referring to an object in an object-oriented programming language, the object's fields and methods are available to the user immediately, through the object's interface. To mimic an object in a relational database, the database must be queried for all attributes of interest, and each value must be accessed separately.
- Objects may contain methods. For example, a person object may contain a print() method, which outputs all information related to the objects in a suitable format. The client programmer, who builds the user interface, does not have to worry how this information is gathered. This programmer only needs to grab the information and present it in a nice layout on a form. Furthermore, different subtypes of the person class, eg, patient or doctor, may have different implementations of the print() method. This is an example of polymorphism and is one of the most powerful features of object-oriented programming languages.
- Classes may be reused. If a class has been designed, it may be reused in other applications; and if a class is redesigned (eg, to improve execution speed) the client programmer does not need to know this, as long as the class' interface is unchanged.

A detailed discussion of object-oriented programming is outside the scope of this article. However, the power of object-oriented programming may be summarized in the terms *encapsulation* and *polymorphism*. Encapsulation means that an object knows all about itself and that it interacts with the surroundings only through a well-defined interface. Encapsulation facilitates reuse and safe programming. Polymorphism means "having many forms." A polymorphic reference is one that can refer to objects of different (sub) types at different times, which is exactly what we need in a generic database.

It is obvious that these (and other) facilities of object-oriented programming languages would be of immense value in the creation of generic clinical databases. It is, however, important to realize that a database management system, whether object-oriented or not, comprises much more than a programming and query language—important issues being storage management, transaction management and concurrency control—and these issues are still under development in object-oriented database management systems. (Concurrency control involves locking parts of the database to prevent unintentional overwriting of data.)

### Conclusions

The objective of generic database design is to provide a robust physical database schema that does not need to change as the domain evolves. Generic databases are of special interest for clinical information systems, and several approaches to generic design have been exercised. They have in common the use of Entity-Attribute-Value tables for storing data and a number of metadata tables to describe the semantics and the relations between data. An object-oriented approach to generic modeling of metadata is by far the most flexible and domain-independent approach. However, the overhead in taking this approach may not be justified for less-advanced applications.

Further studies regarding the implementation of object-oriented database management systems for the purpose of generic clinical databases are suggested.

## Abbreviations

ACT/DB: Adaptable Clinical Trials DataBase
CPMC: The Clinical Data Repository at Columbia-Presbyterian Medical Center
EAV: Entity-Attribute-Value
EAV/CR: EAV with Classes and Relations
SQL: Structured Query Language
